# [6]-Gingerol induces Caspase-Dependent Apoptosis in Bladder Cancer cells via MAPK and ROS Signaling

**DOI:** 10.7150/ijms.73077

**Published:** 2022-06-21

**Authors:** Na Ri Choi, Woo-gyun Choi, Min Ji Kwon, Joo Han Woo, Byung Joo Kim

**Affiliations:** 1 Division of Longevity and Biofunctional Medicine, Pusan National University School of Korean Medicine, Yangsan 50612, Republic of Korea.; 2 Department of Physiology, Dongguk University College of Medicine, Gyeongju, 38066. Republic of Korea.; 3 Channelopathy Research Center (CRC), Dongguk University College of Medicine, 32 Dongguk-ro, Ilsan Dong-gu, Goyang, Gyeonggi-do, 10326. Republic of Korea.

**Keywords:** [6]-gingerol, cell proliferation, apoptosis, bladder cancer, 5637

## Abstract

The anti-cancer effects of [6]-gingerol ([6]-GIN), the main active polyphenol of ginger (*Zingiber officinale*), were investigated in the human bladder cancer cell line 5637. [6]-GIN inhibited cell proliferation, increased sub‑G1 phase ratios, and depolarized mitochondrial membrane potential. [6]-GIN-induced cell death was associated with the downregulation of B‑cell lymphoma 2 (BCL‑2) and survivin and the upregulation of Bcl‑2‑associated X protein (Bax). [6]-GIN activated caspase‑3 and caspase-9 and regulated the activation of mitogen-activated protein kinases (MAPKs). Further, [6]-GIN also increased the intracellular reactive oxygen species (ROS) levels and TG100-115 or tranilast increased [6]-GIN‑induced cell death. These results suggest that [6]-GIN induced apoptosis in the bladder cancer cell line 5637 and therefore has the potential to be used in the development of new drugs for bladder cancer treatment.

## Introduction

Bladder cancer is one of the most frequent genitourinary malignant neoplasms 1 and non-muscle-invasive bladder cancer is the most common type of bladder cancer, accounting for 75% of the cases 2. Currently, after surgery to remove bladder cancer, the remaining micro cancer tissues are treated with chemotherapy or radiation to prevent recurrence 3,4. However, studies report that only a few of the current chemotherapy methods are effective in preventing the recurrence of bladder cancer 5. Also, serious complications are known to occur 6. Hence, more effective strategies are required for successfully treating bladder cancer.

The ginger rhizomes (*Zingiber officinale*) are among the most frequently used medicinal herbs 7. The anti-inflammatory, anti-obesity, antiemetic, antioxidant, antibacterial, and anticancer effects of ginger have been studied 8,9. Ginger contains 6-gingerol (6-GIN), 6-shogaol, 6-paradol, and zingerones 10. Among these components, 6-GIN (1-[49-hydroxy-39-methoxyphenyl]-5-hydroxy-3-decanone) has been known to exhibit anticancer efficacy in various cancer cell lines 11-15.

Apoptosis is the cell's natural mechanism for programmed cell death and is a promising target for anticancer therapy 16. Hence, the prevention of cancer is one of the main functions of apoptosis 17. However, the anti-cancer efficacy and related mechanisms of 6-GIN in bladder cancer are not well understod. In the present study, we investigated the anticancer mechanisms of 6-GIN in the human bladder cancer cell line 5637.

## Materials and Methods

### Reagents and cell culture

6-GIN was purchased from Sigma-Aldrich (St. Louis, MO, USA). Human urinary bladder carcinoma cell line 5637 was obtained from the Korean Cell Line Bank (Seoul, Korea). The cells were propagated in RPMI-1640 medium (Gibco-BRL, St. Louis, MO, USA) supplemented with 10% heat-inactivated fetal bovine serum (Invitrogen, Grand Island, NY, USA) containing 1% penicillin/streptomycin (Invitrogen), and incubated in a humidified 95% air and 5% CO_2_ atmosphere at 37 °C.

### MTT assay

Cell viability was determined using an MTT (3-[4,5-dimethylthiazol-2-yl]-2,5-diphenyltetrazolium bromide) assay kit (Sigma-Aldrich). 5637 cells were treated with MTT solution and incubated for 2 h at 37 °C, following which, absorbance was measured at 570 nm.

### Cell cycle analysis

5637 cells were treated with ethyl alcohol and vortexed before overnight incubation at 4 ˚C. Samples were centrifuged for 5 min and cell pellets were resuspended in propidium iodide staining solution containing RNase (2 μL), and recentrifuged at 20000 × *g* for 10 s. Samples were analyzed using a fluorescence-activated cell sorter (FACScan; Becton-Dickinson, Mountain View, CA, USA) at λ = 488 nm using Cell-Quest software (Becton-Dickinson, Franklin Lakes, NJ, USA).

### Measurement of mitochondrial depolarization assay

Cells were treated with 50 nM tetramethylrhodamine methyl ester (TMRM; Sigma-Aldrich) for 30 min. Fluorescence intensities were measured using a BD FACSCANTO II (BD Biosciences, Sunnyvale, CA, USA) at the excitation and emission wavelengths of 510 and 580 nm, respectively.

### Western blot analysis

Cell lysates were prepared using RIPA buffer containing a protease and phosphatase inhibitor cocktail (Calbiochem, La Jolla, CA, USA). The total protein was quantified using Bradford assay (Bio-Rad Laboratories, Hercules, CA, USA). Equal amounts of protein (20 μg per lane) from the samples were separated by 8% or 10% SDS-PAGE and probed with the indicated antibodies. Antibodies against survivin (#2808), ERK (#9102), pERK (#9106), JNK (#9252), pJNK (#9251), p38 (#9212), and pp38 (#9216) were purchased from Cell Signaling Technology (Danvers, MA, USA), and antibodies against BCl (#sc-783), Bax (#sc-493), caspase-3 (#sc-7148), caspase-9 (#sc-7885), PARP (#sc-7150), β-actin (#sc-47778) and GAPDH (#sc-32233) were obtained from Santa Cruz Biotechnology (Santa Cruz, CA, USA). The secondary horseradish peroxidase-conjugated antibodies used were goat anti‑rabbit IgG and goat anti‑mouse IgG (SC‑2004 and SC-2005, respectively; Santa Cruz Biotechnology, Dallas, TX, USA). Relative intensities of the bands were analyzed with a GS‑710 Image Densitometer (Bio‑Rad Laboratories). Results are representative of at least five independent experiments.

### Caspase assay

Caspase-3 and caspase-9 assay kits (Cellular Activity Assay Kit Plus; BioMol, Plymouth, PA, USA) were used. After resuspending the cells in ice‑cold cell lysis buffer, the supernatant samples were incubated with a caspase substrate (400‑lM Ac‑DEVD‑pNA; 50 μL) at 37 °C. The fluorescence of each sample was measured at 405 nm at several time‑points.

### Measurement of ROS levels

Cells were treated with 20 μL DCF‑DA (2′,7′-dichlorodihydrofluorescein diacetate; Molecular Probes, Eugene, OR, USA) at 37 °C for 30 min and washed with PBS. Fluorescence was measured using FACS (Becton-Dickinson, Mountain View, CA, USA), at excitation and emission wavelengths of 488 and 525 nm, respectively.

### Statistical analysis

One-way ANOVA with *Dunnett's* post hoc comparison was used for multiple comparisons. The analysis was performed using Prism 6.0 (GraphPad Software Inc., La Jolla, CA, USA) and Origin 8.0 (OriginLab Corporation, Northampton, MA, USA) software. Results are expressed as means ± SEMs, and *p* values < 0.05 were considered statistically significant.

## Results

### Apoptotic effects of 6-GIN on 5637 bladder cancer cells

**Figure 1:**
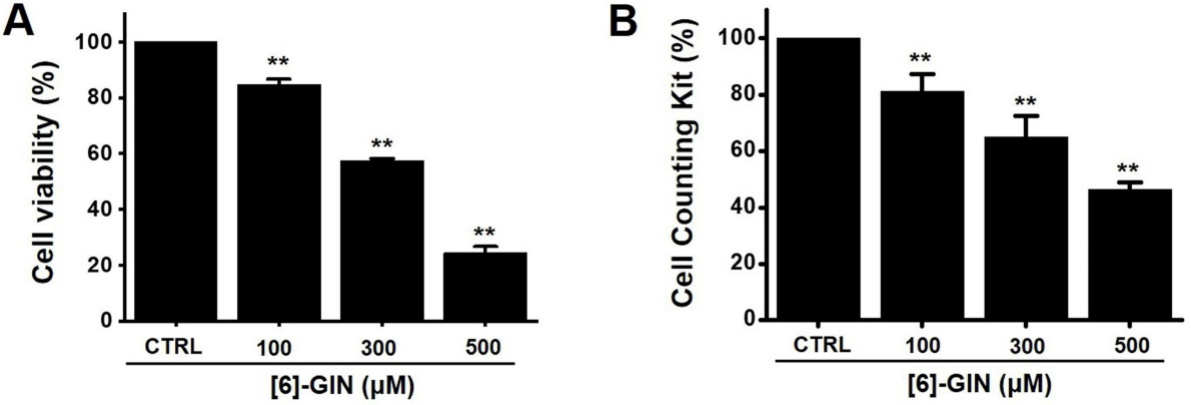
6-GIN reduced the cell viability of 5637 bladder cancer cells measured using MTT assay. 6-GIN reduced cell viabilities dose-dependently after 24 h (**A**). (**B**) CCK-8 (cell counting kit-8) assays showing 6-GIN reduced cell viability after 24 h. Bars represent mean ± standard error. **p <0.01 compared with the control. 6-GIN, 6-gingerol. CTRL, control.

**Figure 2:**
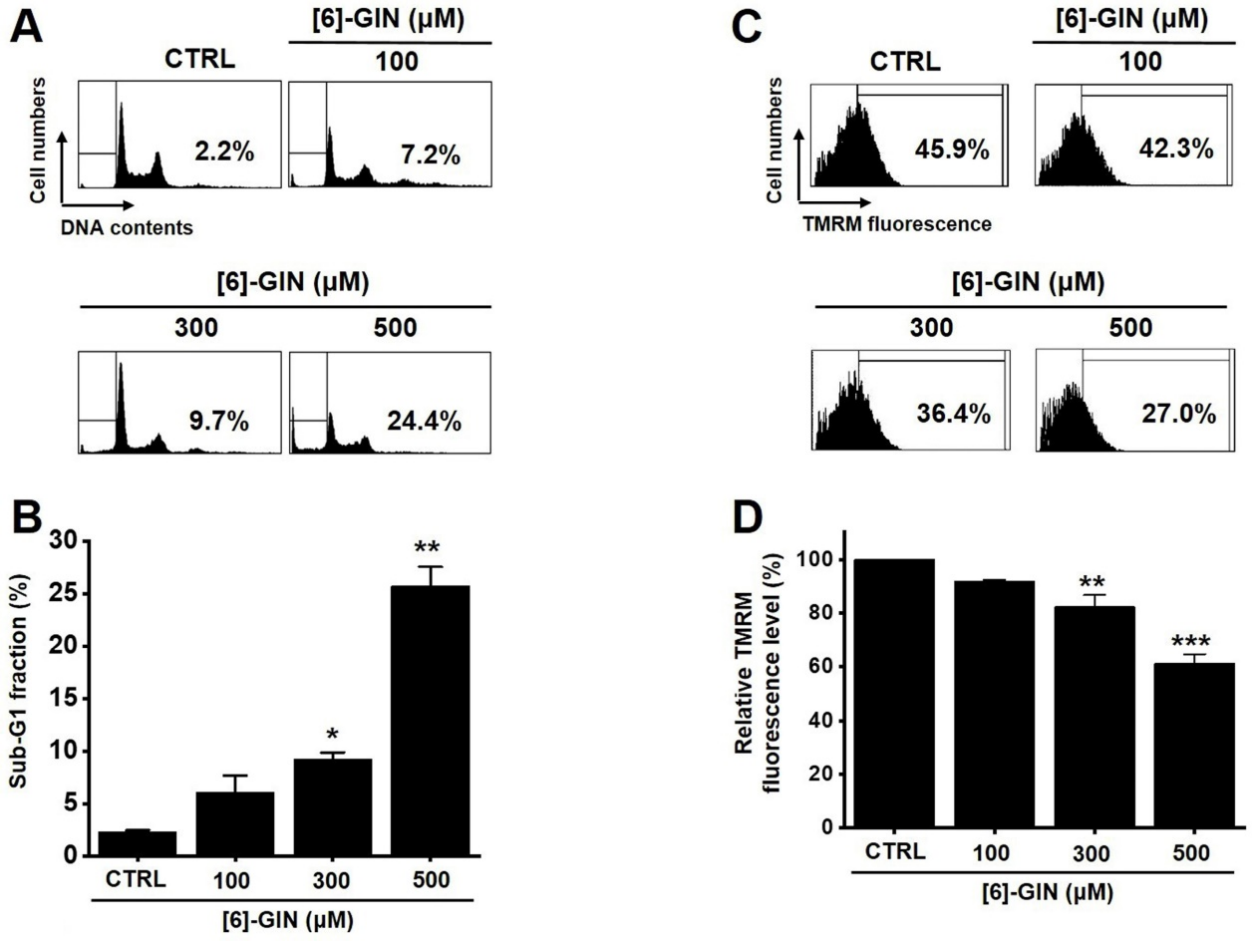
6-GIN increased the sub‑G1 phase ratio and depolarized the mitochondrial membrane of 5637 cells. **(A)** Cell cycles determined with flow cytometry. **(B)** Sub-G1 fractions are expressed as percentages. **(C)** FACS analysis measured the TMRM fluorescence for mitochondrial depolarization. **(D)** The relative mitochondrial TMRM fluorescence levels were calculated. Bars represent mean ± standard error. *p <0.05. **p <0.01. ***p <0.01 compared with the control. 6-GIN, 6-gingerol. CTRL, control. TMRM, tetramethylrhodamine methyl ester.

The MTT assay was used after treating 5637 cells with 6-GIN for 24 h to determine the effect of 6-GIN on the proliferation of the cells. 6-GIN (100, 300, or 500 µM) treatment reduced the survival of 5637 cells in a concentration and time-dependent manner (Fig. 1A). Following 24 h of treatment, the survival of 5637 cells was reduced by 84.9 ± 1.9%, 57.6 ± 0.7%, and 24.5 ± 2.2% (all *p* < 0.01) with 100, 300, and 500 μM of 6-GIN, respectively (Fig. 1A). The survival of 5637 cells was also evaluated using the CCK method. 6-GIN also reduced the viability of the cells by 81.3 ± 6.2%, 64.9 ± 7.4%, and 46.3 ± 2.5% (all *p* < 0.01) at concentrations of 100, 300, and 500 μM, respectively (Fig. 1B). Additionally, cell cycle analysis was conducted using flow cytometry to determine whether 6-GIN induced apoptosis. Sub-G1 phase ratios were increased by 6.1 ± 1.6 %, 9.3 ± 0.6 % (*p* < 0.05), and 25.7 ± 1.8 % (*p* < 0.01) following treatment with 100, 300, and 500 µM 6-GIN, respectively, when compared with thevalues obtained for the non-treated cells (Fig. 2A and 2B). Mitochondrial depolarization was assessed by TMRM staining and the results showed that the mitochondrial membrane of the cells was significantly depolarized by 6-GIN (Fig. 2C). Relative TMRM fluorescence levels were decreased by 91.8 ± 0.6%, 82.4 ± 4.4% (*p* < 0.01), and 61.3 ± 3.5% (*p* < 0.001) following treatment with 100, 300, and 500 µM 6-GIN, when compared with the values observed for the non-treated cells (Fig. 2D). These results suggested that 6-GIN inhibited the proliferation of 5637 cells and these effects could be associated with apoptotic cell death.

### 6-GIN-induced apoptosis via the mitochondrial activation mechanism

**Figure 3:**
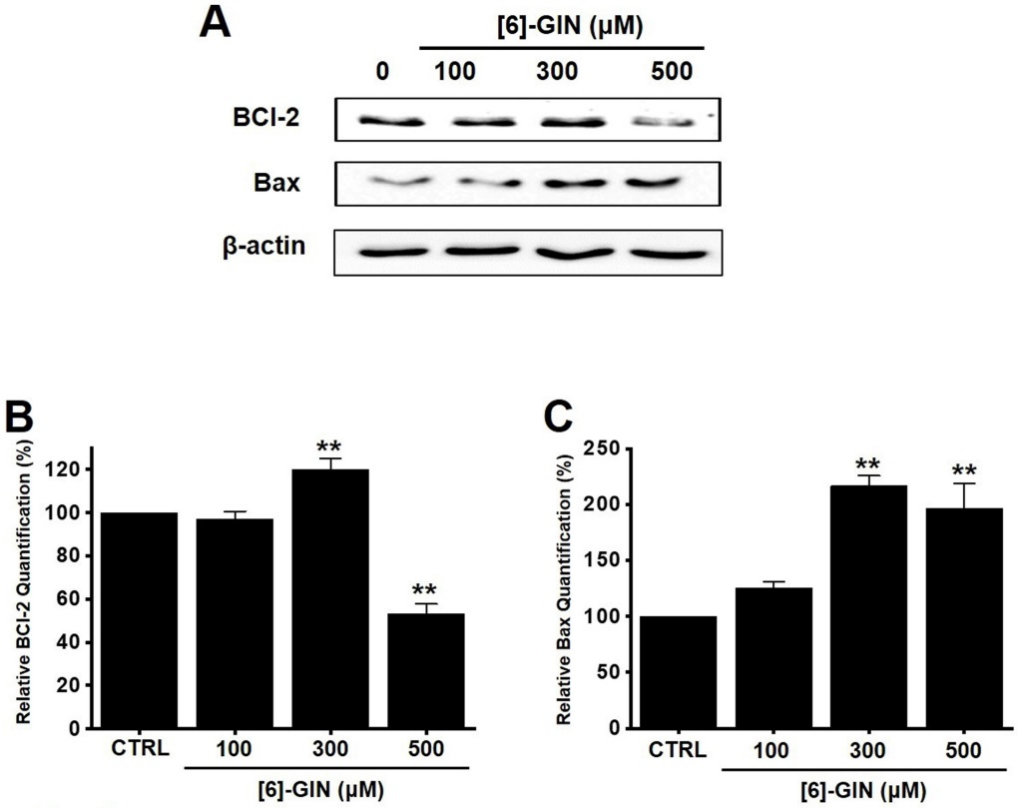
Effects of 6-GIN on BCL-2 and Bax expression in 5637 cells determined by western blotting (**A**) BCL-2 expression was downregulated, whereas Bax was upregulated by 6-GIN (**B**) BCL‑2 and (**C**) Bax protein expressions were normalized versus β‑actin. Bars represent mean ± standard error. **p <0.01 compared with the control. 6-GIN, 6-gingerol. CTRL, control. BCl-2, B‑cell lymphoma 2. Bax, BCL‑2 X‑associated protein.

**Figure 4:**
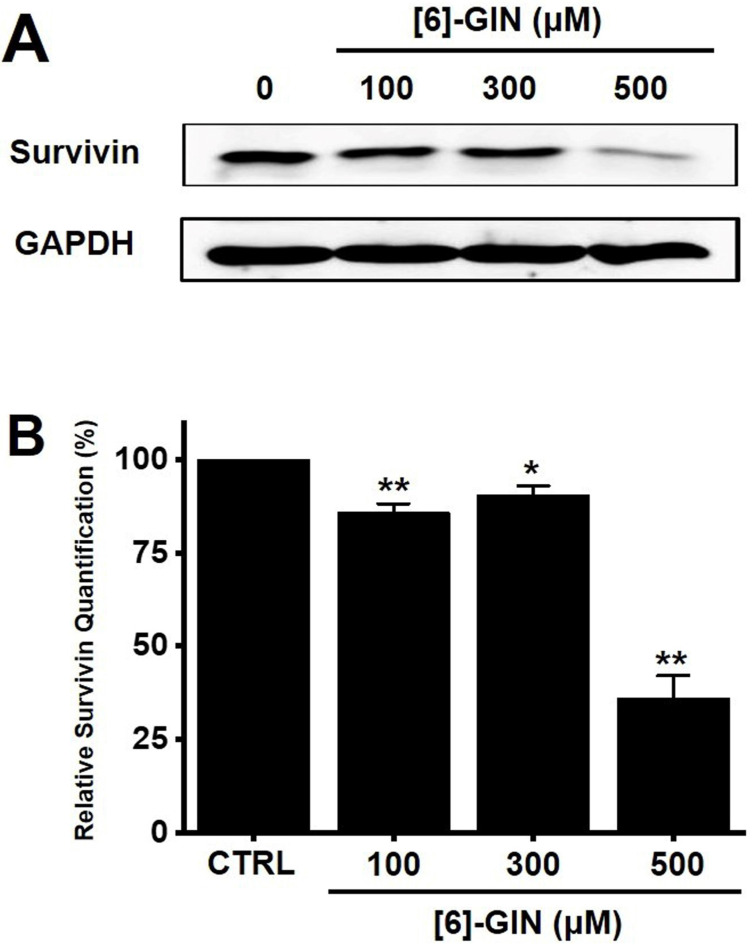
Effects of 6-GIN on survivin expression in 5637 cells determined by western blotting. **(A)** Survivin expression was downregulated by 6-GIN **(B)** Survivin protein expressions were normalized versus β‑actin. Bars represent mean ± standard error. *p <0.05. **p <0.01 compared with the control. 6-GIN, 6-gingerol. CTRL, control.

Western blotting was performed to determine whether the 6-GIN-induced apoptosis is regulated by the BCL-2 (anti-apoptotic) and Bax (pro-apoptotic) proteins. BCL-2 levels were reduced by 6-GIN addition, whereas the amount of Bax increased (Fig. 3A, 3B, 3C). In 5637 cells, compared with non-treated cells, relative BCL-2 levels were decreased by 97.2 ± 3.4%, 119.9 ± 5.2% (*p* < 0.01), and 53.3 ± 4.4% (*p* < 0.01) following treatment with 100, 300, and 500 µM of 6-GIN, respectively (Fig. 3B); however, the relative Bax levels were increased by 124.9 ± 6.2%, 216.8 ± 9.0% (*p* < 0.01), and 196.7 ± 22.3% (*p* < 0.01) upon treatment with 100, 300, and 500 µM 6-GIN, respectively (Fig. 3C). Expression of survivin, a member of the family of apoptosis inhibitor proteins, was also reduced by 6-GIN (Fig. 4A, 4B). Relative survivin quantification levels were decreased by 85.9 ± 2.3% (*p* < 0.01), 90.6 ± 2.3% (*p* < 0.05), and 36.2 ± 5.9% (*p* < 0.01) at 100, 300, 500 µM 6-GIN, respectively, when compared with the levels observed in the non-treated cells (Fig. 4B). These results suggest that mitochondrial activation mechanisms are involved in the 6-GIN-induced apoptosis of 5637 cells.

### 6-GIN-induced apoptosis via caspase activation mechanism

**Figure 5:**
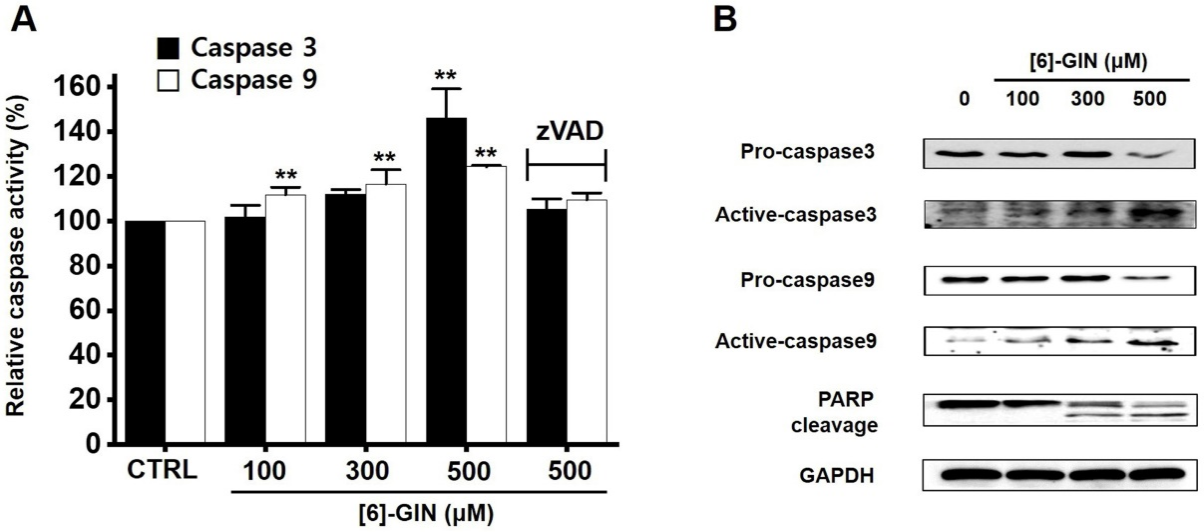
6-GIN-induced caspase activation in 5637 cells*.* **(A)** 6-GIN increased the caspase-3 and caspase-9 activities. **(B)** Western blot analysis showed that 6-GIN gradually down-regulated the expressions pro-caspase-3 and -9 and upregulated the active caspase-3, -9, and PARP cleavage protein levels. GAPDH was used as the internal control. Bars represent mean ± standard error. **p <0.01 compared with the control. 6-GIN, 6-gingerol. CTRL, control. zVAD, carbobenzoxy-valyl-alanyl-aspartyl-[O-methyl]- fluoromethylketone.

Caspases are important mediators in apoptotic pathways. They induce the activation of cytoplasmic endonucleases, which cleave various substrates, such as poly (ADP‑ribose) polymerase (PARP) as a signal for apoptosis 18. 6-GIN addition increased the activities of caspase-3 and caspase-9 in a dose-dependent manner, and zVAD-fmk (a broad-spectrum caspase inhibitor) suppressed these activities (Fig. 5A). Compared with the non-treated cells, in 5637 cells, caspase-3 activity was increased by 102.1 ± 5.1%, 112.1 ± 2.1%, and 146.2 ± 13.0% (*p* < 0.01) with 100, 300, and 500 µM of 6-GIN, respectively, and the caspase-9 activity was increased by 111.9 ± 3.3% (*p* < 0.01), 116.5 ± 6.4% (*p* < 0.01), 124.3 ± 0.6 % (*p* < 0.01) with 100, 300, and 500 µM of 6-GIN, respectively (Fig. 5A). In addition, western blot results showed that adding 6-GIN gradually downregulated the expression of pro-caspase-3 and pro-caspase-9 and upregulated that of active caspase-3, active caspase-9, and the PARP cleavage protein (Fig. 5B). These results suggest that caspase activation mechanisms may play a role during 6-GIN-induced apoptosis in 5637 cells.

### 6-GIN-induced apoptosis via mitogen-activated protein kinase (MAPK) pathways

**Figure 6:**
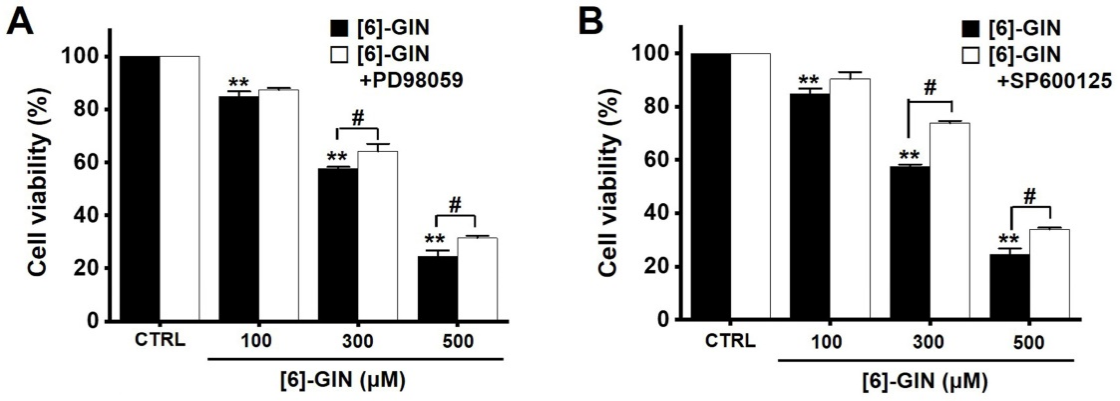
Effects of MAPK inhibitors on the activity of 6-GIN in 5637 cells. Co-administration with 6-GIN and (**A**) PD98059 or (**B**) SP600125 increased cell viability. Bars represent mean ± standard error. **p <0.01 compared with the control. ^#^*p <*0.05 between treatments. 6-GIN, 6-gingerol. CTRL, control.

**Figure 7:**
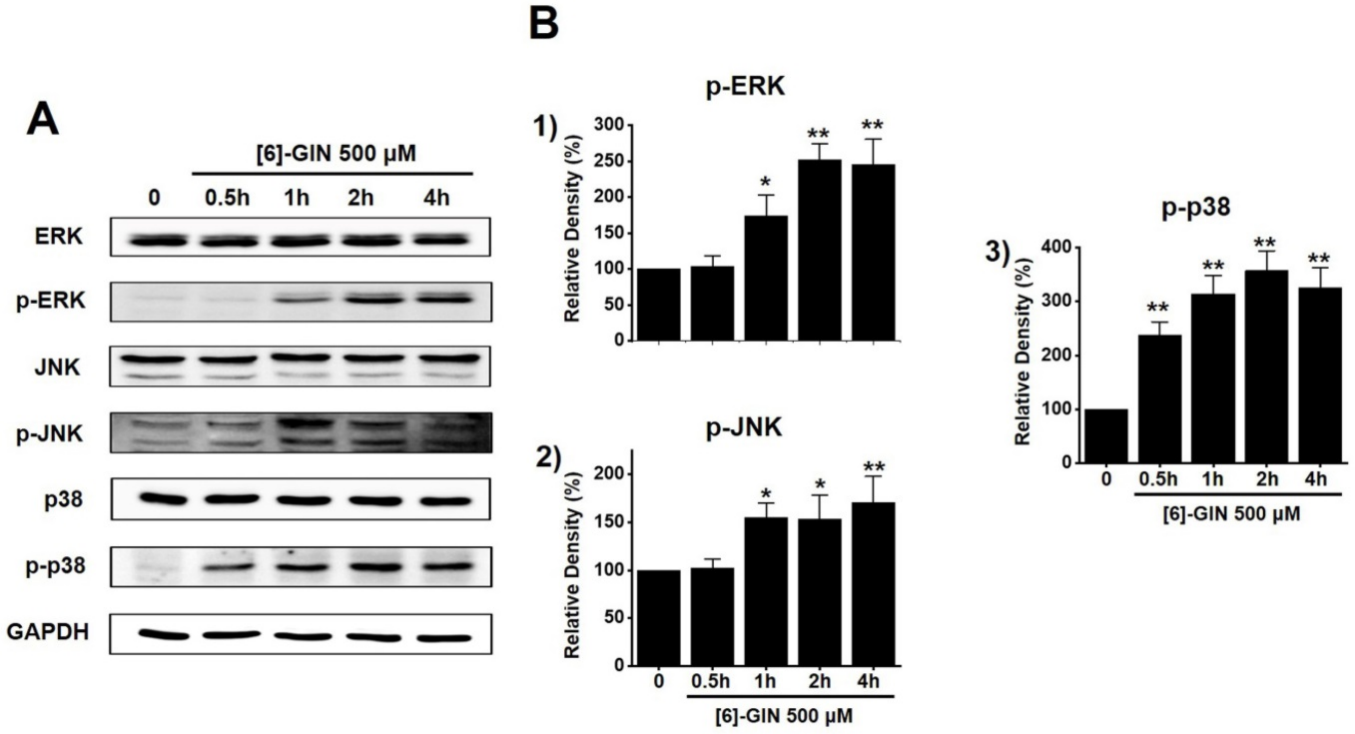
The activation of the MAPK pathways by 6-GIN in 5637 cells. **(A)** Phosphorylation of ERK, JNK, and p38 was increased as observed by western blotting. **(B)** Phosphorylated ERK, JNK, and p38 levels are indicated as band densities relative to GAPDH. Bars represent mean ± standard error. *p <0.05. **p <0.01 compared with the control. 6-GIN, 6-gingerol. CTRL, control. ERK, extracellular signal-regulated kinase; JNK, c‑Jun N‑terminal kinase.

To find out the role of MAPK pathways in 6-GIN-induced apoptosis, cell viability was measured after 6-GIN and PD98059 (p42/44 MAPK inhibitor) or SP600125 (c-jun NH2-terminal kinase (JNK) II inhibitor) were added together. Combination of 6-GIN and PD98059 increased cell viability when compared to non-treated cells (Fig. 6A), and co-administration of 6-GIN and SP600125 increased the viability of the 5637 cells by 90.5 ± 2.6%, 73.9 ± 0.8% (*p* < 0.05), and 34.1 ± 0.6% (*p* < 0.05) at concnetrations of 100, 300, and 500 µM, respectively, compared with the non-treated cells (Fig. 6B). To further examine the involvement of the MAPK signaling pathways, we investigated the 6-GIN-induced phosphorylation of MAPK proteins by western blotting. The phosphorylation of MAPKs increased after treating cells with 6-GIN (500 μM) from 0.5 h to 4 h (Fig. 7A). The levels of phosphorylated extracellular signal regulated kinase (p-ERK) were increased by 103.3 ± 15.3%, 173.7 ± 29.0% (*p* < 0.05), 252.3 ± 22.0% (*p* < 0.01), and 244.7 ± 36.0% (*p* < 0.01) at 0.5, 1, 2, and 4 h, respectively, by 6-GIN treatment, compared with the levels in the non-treated cells (Fig. 7B1); the p-JNK levels were also increased by 102.1 ± 10.0%, 155.2 ± 15.1% (*p* < 0.05), 153.2 ± 25.4 % (*p* < 0.05), and 171.2 ± 27.1% (*p* < 0.01) at 0.5, 1, 2, and 4 h, respectively, by 6-GIN treatment, compared with the levels observed in the non-treated cells (Fig. 7B2). Further, levels of p-p38 were increased by 238.2 ± 23.9%, 314.0 ± 34.1%, 357.1 ± 37.0%, and 326.1 ± 36.3% (all *p* < 0.01) after 0.5, 1, 2, and 4 h of 6-GIN treatment, respectively, compared with the levels in the non-treated cells (Fig. 7B3). These results suggest that MAPK signaling pathways are likely involved in 6-GIN-induced apoptosis.

### 6-GIN-induced apoptosis by intracellular ROS generation

**Figure 8:**
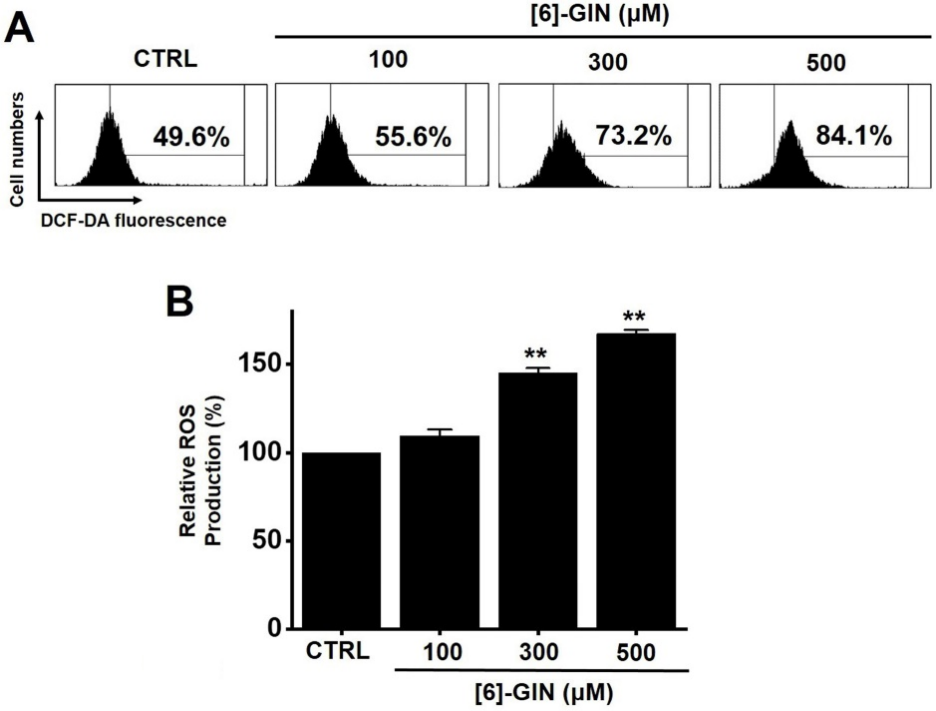
6-GIN increased ROS levels in 5637 cells. **(A)** Intracellular ROS levels were measured by DCF-DA. **(B)** ROS levels are expressed as percentages of untreated CTRL. Bars represent mean ± standard error. **p <0.01 compared to control. 6-GIN, 6-gingerol. CTRL, control. ROS, reactive oxygen species. DCF‑DA, 2',7'-dichlorodihydrofluorescein diacetate.

We investigated whether ROS generation was involved in 6-GIN-induced apoptosis in 5637 cells since ROS are reported to play an important role in apoptosis 19,20. Flow cytometry showed that 6-GIN significantly increased ROS production levels in 5637 cells (Fig. 8A). ROS production levels were increased by 109.5 ± 3.5%, 145.0 ± 2.8% (*p* < 0.01), and 167.5 ± 2.1% (*p* < 0.01) following treatment with 100, 300, and 500 µM of 6-GIN, respectively, compared with the levels in the non-treated cells (Fig. 8B). These results suggest that ROS generation is potentially involved in 6-GIN-induced apoptosis.

### 6-GIN-induced apoptosis via TRPM7 and TRPV2 ion channels

**Figure 9:**
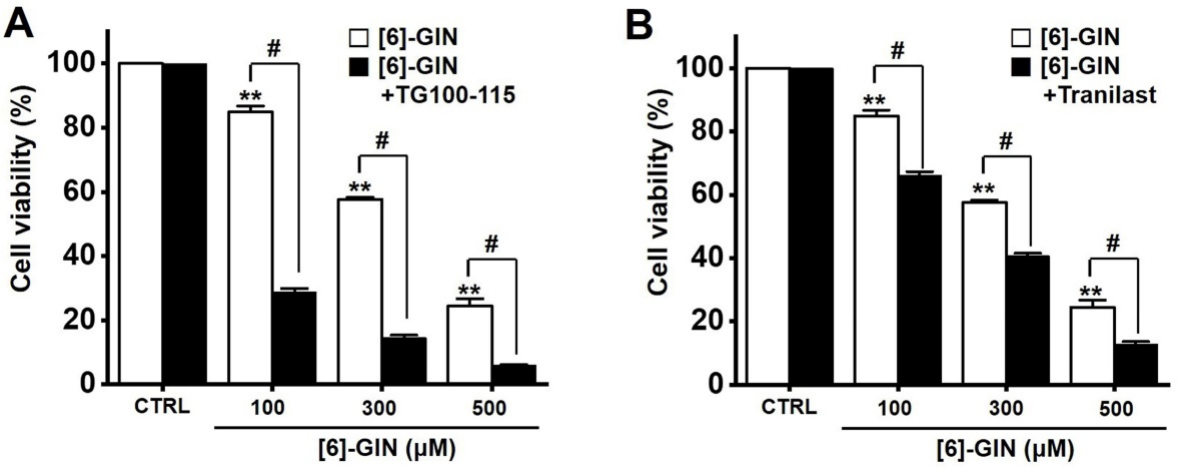
Effects of TG100‑115 or tranilast on 6-GIN-induced apoptosis in 5637 cells. Co-administration for 24 h with 6-GIN and (**A**) TG100-115 or (**B**) Tranilast decreased the cell viability. Bars represent mean ± standard error. **p <0.01 compared to control. ^#^*p* <0.05 between treatments. 6-GIN, 6-gingerol. CTRL, control.

Transient receptor potential melastatin 7 (TRPM7) and vanilloid 2 (TRPV2) are nonselective cation channels that have become attractive target proteins for tumor studies due to their wide range of physiological and pathological functions 21-23. Downregulation of TRPM7 is known to play an important role in bladder cancer cell apoptosis 21,22 and TRPV2 is known to enhance bladder cancer cell migration and invasion but not cell proliferation 23. Therefore, we investigated whether TRPM7 and TRPV2 are involved in 6-GIN-induced apoptosis. Cell viability was measured following co-treatment with 6-GIN and TG100-115 (TRPM7 inhibitor) 24 or tranilast (TRPV2 inhibitor) 25. Compared with the non-treated cells, the co-administration of 6-GIN and TG100-115 decreased the 5637 cell viability by 28.9 ± 1.0%, 14.5 ± 0.8%, and 6.1 ± 0.2% (all *p* < 0.05) at concentrations of 100, 300, and 500 µM, respectively (Fig. 9A), and that of 6-GIN and tranilast decreased the cell viability by 66.2 ± 1.1 %, 40.7 ± 0.8%, and 12.9 ± 0.8% (all *p* < 0.05) at concentrations of 100, 300, and 500 µM, respectively (Fig. 9B). These results suggest that TRPM7 or TRPV2 cation channels are likely involved in 6-GIN-induced apoptosis.

## Discussion

Ginger (*Zingiber officinale*) is one of the most frequently used herbs in traditional Asian medicine 7. It contains pungent phenolic substances such as 6-GIN, 6-shogaol, 6-paradol, and zingerone 10. Among these, 6-GIN is the main active polyphenol of ginger and has been reported to exhibit antioxidant, anti-inflammatory, anticancer, neuroprotective, anti-obesity, and anti-hepatic steatosis effects 26-32. It is also known to have anticancer effects on various cancer cells 11-15, 33-35. 6-GIN has been shown to cause apoptosis in the cervical cancer cell line HeLa by activating the caspase-3-dependent pathway 11. In previous studies, it inhibited the metastasis of the breast cancer cell line MDA-MB-231 and caused the apoptosis of the prostate cancer cell line LNCaP 12,13. 6-GIN also inhibits the growth of several types of murine tumors such as melanomas, renal cell carcinomas, and colon carcinomas 14,15. The present study demonstrates that 6-GIN induces the apoptosis of the bladder cancer cell line 5637.

Apoptosis is a cell's natural mechanism for inducing programmed cell death and is a promising target for anticancer therapy 16. It is caused by caspases which are a class of cysteine proteins that cleave target proteins, and this caspase protease activation is essential for apoptosis to occur 36,37. Four initiator caspases (caspase-2, -8, -9, -10) and three executioner caspases (caspase-3, -6, -7) are known to be involved 37. The intrinsic mechanism of apoptosis requires mitochondria and mitochondrial proteins. We conducted the MTT experiment at various time changes. In the text, cell death can be known according to the concentration at 24 hours (Fig. 1). After 48 hours or 72 hours, it was found that at 100-300 μM, there was less death or no significant change than at 24 hours, and almost all cell death was confirmed at 500 μM. Therefore, it can be seen that 24 hours in the text is the time to confirm the most ideal cell death in a concentration-dependent manner. The overall intrinsic pathway is regulated by the BCL-2 protein family. The anti-apoptotic BCL-2 protein inhibits apoptosis by inhibiting the proapoptotic proteins Bax and BCL-2 homologous antagonist killer 37. In addition, the extrinsic pathway uses extracellular signals to induce apoptosis. Cell death signals bind to death receptors from the tumor necrosis factor (TNF) family 37. In the present study, BCL-2 and survivin levels decreased, while Bax expression was increased by the addition of 6-GIN in 5637 cells (Fig. 3 and 4). The results of 6-GIN-induced BCL downregulation show that 6-GIN can cause apoptosis (Fig. 3). The change in survivin levels further supported this result (Fig. 4). Survivin is known to inhibit apoptosis and combines with the effective caspases of the apoptotic pathway, namely caspase-3 and -7 38,39. 6-GIN increased the relative caspase-3 and caspase-9 activities, and their activities were suppressed by zVAD-fmk (Fig. 5). Moreover, 6-GIN gradually downregulated pro-caspase-3 and -9 and upregulated the active caspases-3 and -9, and PARP cleavage protein expression. These results suggest that 6-GIN induced apoptosis via caspase-dependent death receptor signaling and mitochondrial pathways. Also, we conducted experiments on autophagy. As a result of the experiment, there was no change in the expression of LC3-II. Therefore, it is thought that the autophagy process is not involved in the induction of apoptosis.

MAPKs are a family of kinases that are known to modulate gene expression, mitosis, proliferation, motility, metabolism, and apoptosis 40-44. Particularly, they are involved in the survival and proliferation of various cancer cells, making them potential targets for cancer therapies 45. Similarly, ROS are also essential for cell cycle regulation, differentiation, and migration 46. ROS are important cell signaling molecules involved in the main mechanisms of apoptosis caused by mitochondria 46. ROS are also involved in autophagy and necroptosis 46. In this study, co-administration of 6-GIN and PD98059 or SP600125 increased cell viability (Fig. 6) and the phosphorylation of MAPKs (Fig. 7). Furthermore, 6-GIN increased ROS production levels (Fig. 8). These results suggest that MAPK and ROS signaling pathways are involved in the 6-GIN-induced apoptosis mechanism.

TRP ion channels contribute to regulating various physiological and pathological responses of cells by controlling intracellular signaling and responses 47. Among these, TRPM7 is known to be involved in important physiological processes such as intracellular Mg^2+^ regulation 48, cell viability 49, anoxic neuronal cell death 50, and gastrointestinal motility 51. Recent studies have shown a link between TRPM7 and several cancers such as breast, ovarian, prostate, gastric, bladder, pancreatic cancers, and glioblastomas 52-57. Other TRP ion channels called TRPV channels are critical for normal pain and temperature control 58. TRPV2 channels have many functions, and they may be related to cancer 59,60, particularly urinary tract tumors 61,62. TRPV2 activation induced apoptotic cell death in the T24 bladder cancer cell line 63. In the present study, cell viability was measured after 6-GIN and TG100-115 (TRPM7 inhibitor) or tranilast (TRPV2 inhibitor) were administered together. Co-administration of 6-GIN and TG100-115 or tranilast decreased the cell viability (Fig. 9). These results suggest that TRPM7 or TRPV2 cation channels are involved in 6-GIN-induced apoptosis. However, a potent and dual selective phosphoinositide 3-kinase (PI3K) inhibitor is well known in TG100-115 64 and is also found to be effective as a TRPM7 inhibitor 24. Tranilast is known for its efficacy in antiallergic function and inhibitor of angiogenesis 65,66 and its function as a TRPV2 inhibitor has also been revealed 25. There are always various effects when researching using inhibitors, so be careful when you think about the results. Therefore, we think that more in-depth research is needed such as using various inhibitors to compare results with each other. TG100-115 also has the function of PI3Kγ/δ inhibitor 67. Therefore, we checked the relevance of this function. We used GDC-0032 with the function of PI3Kγ/δ inhibitor 68. As a result of the experiment, some apoptosis was observed in the presence of GDC-0032, but no apoptosis to the extent of TG100-115. Therefore, the reaction by TG100-115 is considered to be the result obtained by inhibiting TRPM7 rather than the reaction by PI3Kγ/δ inhibition. Several TRP ion channels are associated with cancer apoptosis and are potential therapeutic targets 69. The most representative TRP ion channel is TRPM8. TRPM8 is overexpressed in prostate cancer and its expression level is associated with cancer cell death 70. At the same time, menthol, a TRPM8 activator, inhibits the proliferation of prostate cancer cell lines. Because patients with chronic prostatic hyperplasia often develop prostate cancer, they may benefit from TRPM8 activator therapy 71. Therefore, in this study, the relevance of TRPM7 or TRPV2 was mentioned. In the future, the possibility of other ion channels such as TRPM8 will be further studied.

In conclusion, the present study shows that 6-GIN inhibits cell proliferation, increases sub‑G1 phase ratios, and depolarizes mitochondrial membrane potential in 5637 bladder cancer cells. 6-GIN‑induced apoptosis of cancer cells was associated with proteins such as BCL‑2, survivin, Bax, caspase‑3 and caspase-9, and MAPK. Intracellular ROS levels regulate the 6-GIN‑induced cell death and TG100-115 or tranilast had an effect of enhancing the 6-GIN‑induced cell death. We believe that the results of this study could serve as a basis for the future development of new drugs for bladder cancer treatment.
